# Moss-inhabiting flea beetles in the Philippines (Coleoptera, Chrysomelidae, Alticinae)

**DOI:** 10.3897/zookeys.960.54011

**Published:** 2020-08-17

**Authors:** Albert F. Damaška, Dale Joy Mohagan, Martin Fikáček

**Affiliations:** 1 Department of Zoology, Faculty of Science, Charles University, Prague, Czech Republic Charles University Prague Czech Republic; 2 Biology Department, Central Mindanao University, University Town, Maramag, 8710 Bukidnon, Philippines Central Mindanao University Bukidnon Philippines; 3 Department of Entomology, National Museum, Prague, Czech Republic National Museum Prague Czech Republic

**Keywords:** Chrysomelidae, Coleoptera, moss-inhabiting flea beetles, new combination, new species, Philippines, taxonomy

## Abstract

The Philippine islands are one of the key biodiversity hotspots in the Indo-Pacific area. Knowledge of moss-inhabiting flea beetles (Coleoptera: Chrysomelidae: Alticinae), a diverse and ecologically and morphologically enigmatic group in the Philippines is described. Six species from the Philippines are recorded, belonging to three genera: *Benedictus luzonicus* Sprecher-Uebersax et al., 2009 (recorded from the Philippines previously), *Ivalia antennata***sp. nov.**, *I. caligulata***sp. nov.** and *I. postfasciata* (Chen, 1934), **comb. nov.** (transferred from *Chabria* Jacoby, 1887), *Cangshanaltica mindanaoensis***sp. nov.**, and *C. luzonica***sp. nov.** Cox1 barcode sequences of *Ivalia antennata* and *Cangshanaltica mindanaoensis* are presented. Biogeography and diversity of moss-inhabiting flea beetles in the Philippines are discussed.

## Introduction

The Philippine archipelago is one of the world’s biodiversity hotspots (Myers et al. 2000). It is characterised by a large number of species and a high level of endemism. The high level of endemism is a consequence of a unique and complex geological history of the archipelago and its current high habitat diversity, although many habitats are under heavy human exploitation. High endemism is underpinned by the presence of high-mountain sky islands, increasing isolation of populations within and between real islands. These unique, highly isolated, high-altitude areas host many specialised organisms. One of them are moss-inhabiting flea beetles, a specific ecological group of flea beetles (Chrysomelidae: Alticinae) containing many non-related genera worldwide (Konstantinov and Konstantinova 2011, Konstantinov et al. 2013, Ruan et al. 2017). In the Oriental region, the majority of moss-inhabiting flea beetle species diversity is represented by three genera, *Ivalia* Jacoby, 1887, *Benedictus* Scherer, 1969, and *Cangshanaltica* Konstantinov et al., 2013. A single species, *Benedictus luzonicus* Sprecher-Uebersax et al., 2009, is known from the Philippines, contrasting to the high species diversity of other Philippine insect genera. In this study, we show that moss-inhabiting flea beetle fauna is much more diverse in the Philippines. Our review of available material, including the specimens collected during our recent fieldwork, reveals the occurrence of six species, of which five are new to science. In addition to the morphological study of the material, we are also providing cox1 barcodes for the species available in DNA grade, including the first genetic data for *Cangshanaltica*.

## Material and methods.

We examined specimens from the museum collections listed below, as well as those collected during the biodiversity survey performed by us in southern Mindanao in 2017. Samples were collected by sifting moss and the surrounding leaf litter in montane cloud forests; specimens were extracted from the samples by AFD and Matyáš Hiřman. Most specimens were dissected for genitalia examination and mounted on mounting cards. Genitalia were mounted on a separate mounting card, embedded in the water-soluble dimethyl hydantoin formaldehyde (DMHF) resin. Photographs were taken by Canon EOS 550D or 70D camera equipped with the Canon MP-E 65 mm f/2.8 1–5× lens, and using an Olympus BX40 microscope. The morphological terminology follows Lawrence et al. (2010); terminology of head structures follows on Ruan et al. (2019).

The complete DNA was extracted by Qiagen DNEasy Blood and Tissue kit or GenAid Genomic DNA Mini kit. Due to the very small size of the specimens, incubation in proteinase K and tissue lysis were conducted in a thermo-shaker, and 50 μl of elution buffer was used in the final step. For better DNA yield and for cleaning the genitalia by proteinase K before dissection, the body wall of the specimens was perforated before the DNA extraction by breaking abdominal tergites. DNA extracts are stored deep-frozen at the Faculty of Science, Charles University, Prague. For PCR reactions, we used a modified protocol with a commercially prepared premix (PPP Mix with MgCl_2_ added, Top-Bio Czech Republic). We used standard *cox1* barcode primers: forward LCO1490 (5‘-GGTCAACAAATCATAAAGATATTGG-3‘) and reverse HCO2198 (5‘-TAAACTTCAGGGTGACCAAAAAATCA-3‘) (Folmer et al. 1994). PCR was performed in a 13 μl total volume of the mixture, containing 6.25 μl of PPP Mix, 4.75 μl of PCR ddH_2_O, 1.0 μl of each primer and 1.0 μl of the DNA extract. The following PCR program was used: 94 °C for 180 seconds + 35× (94 °C for 30 seconds, 48 °C for 45 seconds, 72 °C for 60 seconds) + 72 °C for 480 seconds. PCR products were purified by adding 0.5 μl Exonuclease 1 [Exo1 (20 U/μL)] (ThermoFisherScientific) and 1.0 μL Thermosensitive Alkaline Phosphatase [FastAP (1 U/μL)] (ThermoFisherScientific); the mixture was incubated in a thermocycler for 37 °C for 15 minutes and 80 °C for 15 minutes. Samples were sequenced by using Sanger sequencing. Raw sequence data were edited by using Geneious 9.1.7 software (Biomatters). Sequences were submitted to GenBank under accession numbers MT654528 and MT654527.

Examined specimens are deposited in the following collections:

**ADPC** Albert F. Damaška personal collection, Prague, Czech Republic;

**IZCAS**Institute of Zoology, Chinese Academy of Sciences, Beijing, China (Ming Bai, RuiE Nie);

**MHNG** Muséum d‘Histoire Naturelle, Geneva, Switzerland (Giulio Cuccodoro);

**NMPC**Department of Entomology, National Museum, Prague, Czech Republic (Lukáš Sekerka).

## Results

### Cangshanaltica

Taxon classificationAnimaliaColeopteraChrysomelidae

Konstantinov, Chamorro, Prathapan, Ge & Yang, 2013

10698C89-08BB-567E-B685-A9F6B68A2B09

#### Type species.

*Cangshanaltica nigra* Konstantinov, Chamorro, Prathapan, Ge & Yang, 2013

#### Type locality.

Yunnan, Dali, Cangshan Mt.

#### Remarks.

The genus *Cangshanaltica* is known to be distributed mainly in China and neighbouring areas (Damaška and Aston 2019). Three species have been described so far; descriptions of additional ones are in preparation to date. Here, we describe two new species from Luzon and Mindanao. We place them in this genus based on following characters: (1) round, ovate, and convex body shape; (2) anterior coxal cavities open posteriorly; (3) metatibiae curved in lateral view; (4) metaventrite bearing an anterior, horseshoe-like process reaching mesocoxae and covering the mesoventrite; (5) anterolateral pronotal setiferous pore placed in the middle of the pronotal margin; and (6) antennomere VII bearing a slight distal protrusion.

### Cangshanaltica luzonica sp. nov.

Taxon classificationAnimaliaColeopteraChrysomelidae

95091F46-54AB-5CA5-B55D-9681CE324A95

http://zoobank.org/0FECE94C-83E4-425A-9F87-DB0538104C92

Figures 1A , 3A, B

#### Type locality.

Philippines: Luzon, Sagada env.

#### Type material.

**Holotype** ♂ (MHNG): “Philippines: Luzon. env. Sagada. 15.-19. xii.79, Deharveng-Orousset.”. **Paratypes** (2♀ 1 MHNG, 1 NMPC): same labels as holotype.

#### Differential diagnosis.

The species differs from all known species of *Cangshanaltica* except *C. mindanaoensis* by the presence of metallic elytra. It differs from *C. mindanaoensis* in (1) aedeagus slender, elongate (broad and flattened in *C. mindanaoensis*); (2) elytra nearly impunctate (irregularly punctured in *C. mindanaoensis*); (3) head and pronotum with greenish-bronze lustre, elytra with violet-blue lustre (elytra and pronotum of the same greenish lustre in *C. mindanaoensis*,); (4) male pro- and mesotarsomeres I slender (strongly widened and flattened in *C. mindanaoensis*); (5) tibial spur shorter to as long as metatarsomere II (longer than metatarsomere II in *C. mindanaoensis*).

#### Description.

***Habitus*.** Body round, 2.4–2.6 mm long, 2 mm wide, 1.7 mm high. Head and pronotum black with feeble greenish-bronze metallic lustre, elytra black with violet-blue metallic lustre. Ventral surfaces black, appendages brown to black.

***Head*** nearly hypognathous, triangular in frontal view. Frontal calli nearly indistinct, not surrounded dorsally. Supraorbital, orbital and suprafrontal sulci very deep, supraantennal sulcus forming a deep excavation. Frontal ridge wide, frons with large punctures bearing white setae. Clypeus impunctate, bearing a row of small white setae. Antennae with 11 antennomeres. Antennomere I as long as antennomeres II–III combined, bulbous; antennomere II small, rounded, antennomere III slightly elongated; antennomeres IV–XI gradually widening and slightly elongating, pilose.

***Thorax*.** Pronotum very convex, twice as broad as long. Anterolateral pronotal margin forming a lobe, anterolateral pronotal setiferous pore in the middle of pronotal margin. Posterior pronotal edges dull. Scutellar shield very small, triangular. Elytra strongly convex, nearly impunctate. Metathoracic wings and humeral calli absent. Pro- and mesotibiae densely pilose on ventral side. Mesotibiae and metatibiae slightly curved laterally. Metatarsus attached to metatibia slightly before its end. Metatibial apical spine shorter or as long as metatarsomere II., metatarsomere I 2× longer than metatarsomere II. Metaventral process horseshoe-like, excavated, with dull apex.

***Abdomen*.** Ventrite I bearing an anterior process reaching metacoxae; with a distinct elevated ridge.

***Genitalia*.** Aedeagus strongly sclerotised, slender, elongate, narrowing towards apex in ventral view; slightly curved in lateral view. Apex of aedeagus dull, rounded. Spermatheca small, with long pump and small, rounded receptacle; spermathecal duct placed laterally, forming two loops.

#### Etymology.

The species name refers to the island of Luzon where the type series was collected.

#### Biology.

Unknown.

**Figure 1.**
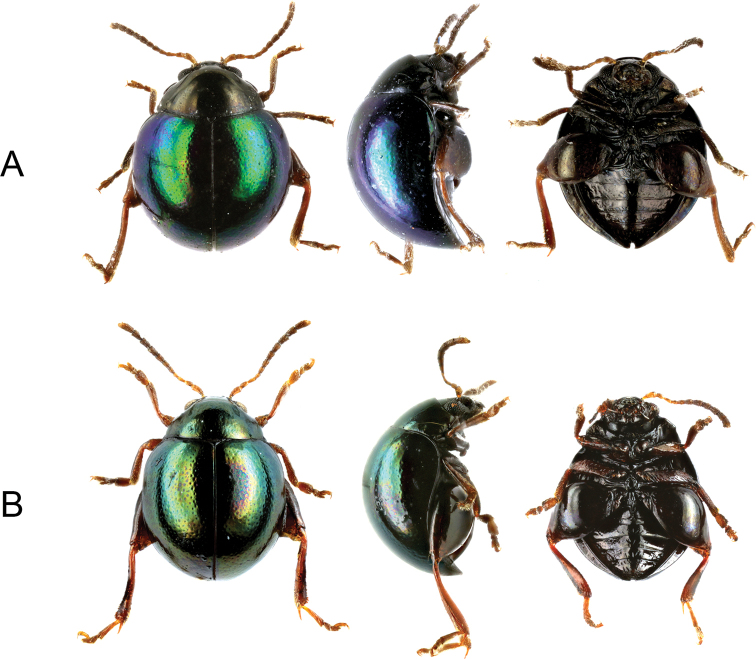
Species of *Cangshanaltica* distributed in the Philippines, dorsal, right lateral, and ventral views **A***C. luzonica* sp. nov. **B***C. mindanaoensis* sp. nov.

### Cangshanaltica mindanaoensis sp. nov.

Taxon classificationAnimaliaColeopteraChrysomelidae

A5A5ED05-D5ED-5D7B-A7ED-F16CC05090E4

http://zoobank.org/E5686EEC-4A44-46AC-ACE5-F0102AA50745

Figures 1B , 3C, D

#### Type locality.

Philippines: Mindanao, Davao Oriental prov., Mt. Hamiguitan.

#### Type material.

***Holotype*** ♂ (NMPC): Philippines – Mindanao, Davao Oriental prov., Mt. Hamiguitan; 6°42'52.7"N, 126°11'38.0"E; sifting montane mossy forest; 20.ii.2017; A. F. Damaška lgt. **Paratypes**: (4♂ 1 NMPC, 2 ADPC, 1 USNM), same labels as holotype.

#### Additional material examined.

1♀ (ADPC): (1) Philippines – Mindanao, Davao City prov., Mt. Malambo, Busay Resort, 1200 m, 7°28'52.83"N, 125°15'43.3"E; sifting montane forest; 23–28.ii.2017, A. Damaška lgt. (2) voucher specimen A. F. Damaška coll., AFD-006.

#### Differential diagnosis.

The species differs from all known species of *Cangshanaltica* except *C. luzonica* by its metallic elytra. For the diagnosis from *C. luzonica*, see the latter species.

#### Description.

***Habitus*.** Body round, strongly convex; 2.2–2.3 mm long, 1.8 mm wide, 1.4 mm high. Dorsal surface generally black with greenish metallic lustre; ventral surfaces black. Legs chestnut brown, metafemora nearly black.

***Head*** nearly hypognathous, widely triangular. Frontal calli wide, slightly surrounded dorsally by shallow sulci. Supraorbital, orbital and suprafrontal sulci distinct, not extremely deep. Frontal ridge wide, feebly elevated; frons bearing two bunches of small punctures on sides; with scattered long setae. Clypeus impunctate, bearing a row of long setae. Antennae with 11 antennomeres. Antennomere I as long as antennomeres II–III combined, bulbous; antennomere II and III equally long; antennomere IV. small, rounded, antennomeres V–XI gradually widening and elongating, pilose.

***Thorax*.** Pronotum strongly convex, twice as broad as long, bearing small sparsely scattered punctures. Scutellar shield small, triangular. Anterolateral pronotal setiferous pore placed in the middle of pronotal margin. Posterior pronotal edges dull. Elytra strongly convex, irregularly punctate. Metatibiae curved laterally. Pro- and mesotarsomeres I in males widened, flat. Metatarsus attached to metatibia slightly before its end. Metatarsomere I strongly elongate, nearly as long as a third of metatibia. Metatibial spur very long, longer than metatarsomere II. Metaventral process horseshoe-like, excavated, with dull apex.

***Abdomen*.** Ventrite I bearing an anterior process reaching metacoxae; with a distinct elevated ridge. Ventrites III–V distinctly punctate.

***Genitalia*.** Aedeagus slightly curved in lateral view; broad in ventral view, with broadly arrow-like apex.

#### Etymology.

The species name refers to the island of Mindanao, where the type series was collected.

#### Biology.

The species was collected in montane cloud forests (Fig. 4) where it inhabits moss cushions.

#### Remarks.

Type specimens were collected on Mt. Hamiguitan, Davao Oriental, Mindanao. The additional female examined was collected in Mt. Malambo, Davao City, Mindanao, ca. 150 km far from Hamiguitan. This female differs slightly from the type series e.g. in the metallic sheen being different between pronotum and elytra – pronotum has less visible, brownish metallic sheen. Unfortunately, we failed to sequence the type specimens and we cannot compare the morphology of genitalia, because all Mt. Hamiguitan specimens are males. Due to these problems and because of strong isolation of montane flea beetle populations, we decided to exclude the specimen from Mt. Malambo from the type series. The spermatheca of this specimen looks as follows: pump slender, receptacle bulbous, spermathecal duct attached posteriorly, orientated anteriorly, without coils. We also can provide the barcode sequence of the female specimen (GenBank accession number MT654527).

### Ivalia

Taxon classificationAnimaliaColeopteraChrysomelidae

Jacoby, 1887

815E0BBA-5299-5868-B241-A0113DDFE324

#### Type species.

*Ivalia viridipennis* Jacoby 1887

#### Type locality.

Sri Lanka

For synonymy, see Duckett et al. (2006).

#### Remarks.

The genus includes 79 known species widespread across the Oriental and Australo-Papuan regions. Large proportions of its species diversity are known from Papua New Guinea and from the Himalayan range (Nadein 2013). A bunch of species was recently described from Borneo (Takizawa and Konstantinov 2018) and two species are described from Taiwan. No species has been recorded from the Philippines. *Ivalia* is diagnosed by having convex, oblong-ovate body shape, anterior coxal cavities open posteriorly, strongly curved metatibiae, usually lacking metathoracic wings and humeral calli, anterolateral pronotal setiferous pore placed in anterior half of the pronotal margin, and metaventrite bearing an anterior, dull horseshoe-like process reaching mesocoxae and partially covering the mesoventrite, a character similar to that in *Cangshanaltica*. However, morphological diversity is relatively wide in species described as *Ivalia*, and many known species lack some of the aforementioned diagnostic characters (e.g., strongly ovate body shape or curved metatibiae). The generic placement of the new species described here is discussed below; the generic assignment needs to be re-confirmed by future phylogenetic studies in the case of *I. caligulata* sp. nov.

### Ivalia antennata sp. nov.

Taxon classificationAnimaliaColeopteraChrysomelidae

7358202D-2D99-5F25-84B8-BC35FDE18BC2

http://zoobank.org/DA9DE777-74C7-4CC7-885D-43AE6B947B3E

Figures 2A , 3E

#### Type locality.

Philippines: Mindanao – Davao City prov., Mt. Malambo.

#### Type material.

Holotype ♂ (NMPC): (1) Philippines – Mindanao, Davao City prov., Mt. Malambo, Busay Resort, 1200 m, 7°28'52.83"N, 125°15'43.3"E; sifting montane forest; 23–28.ii.2017, A. Damaška lgt.; (2) VOUCHER SPECIMEN A. F. Damaška coll., AFD-014.

#### Generic assignment.

The species is assigned to *Ivalia* on the basis of following characters: (1) metathoracic wings and humeral calli absent; (2) body convex and ovate in shape; (3) metatibiae strongly curved laterally; (4) antennomere VII lacking any process; (5) metaventrite reaching mesocoxae and partially covering the mesoventrite with an anterior process.

**Figure 2.**
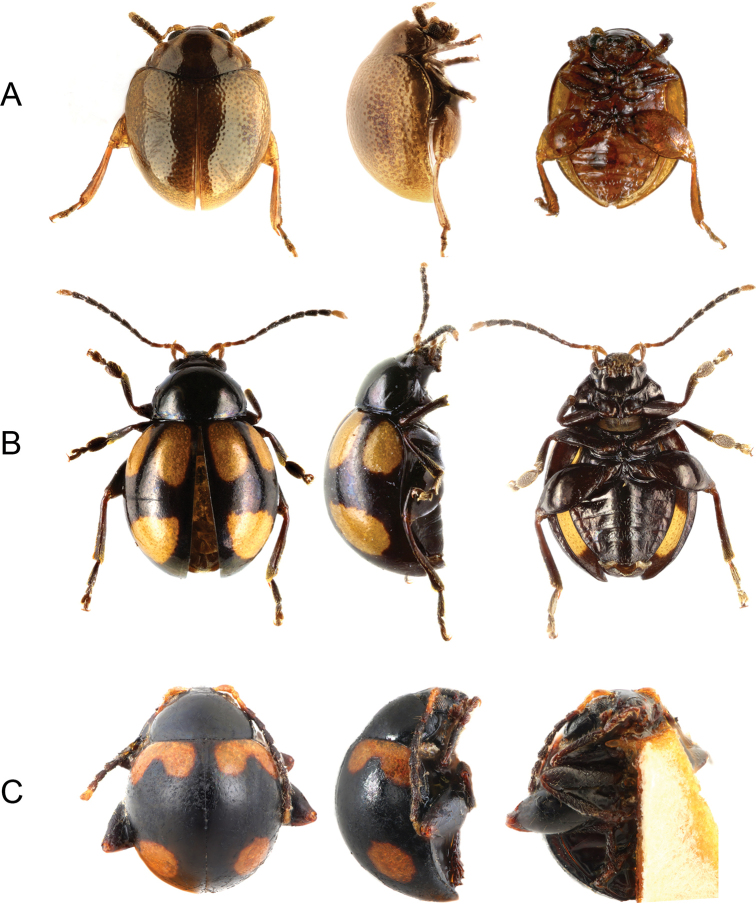
Species of *Ivalia* distributed in the Philippines, dorsal, right lateral, and ventral views **A***I. antennata* sp. nov. **B***I. caligulata* sp. nov. **C***I. postfasciata* (Chen, 1934).

#### Differential diagnosis.

The species differs from all known brown-coloured *Ivalia* species by having an unique, club-like shape of antennae.

**Figure 3.**
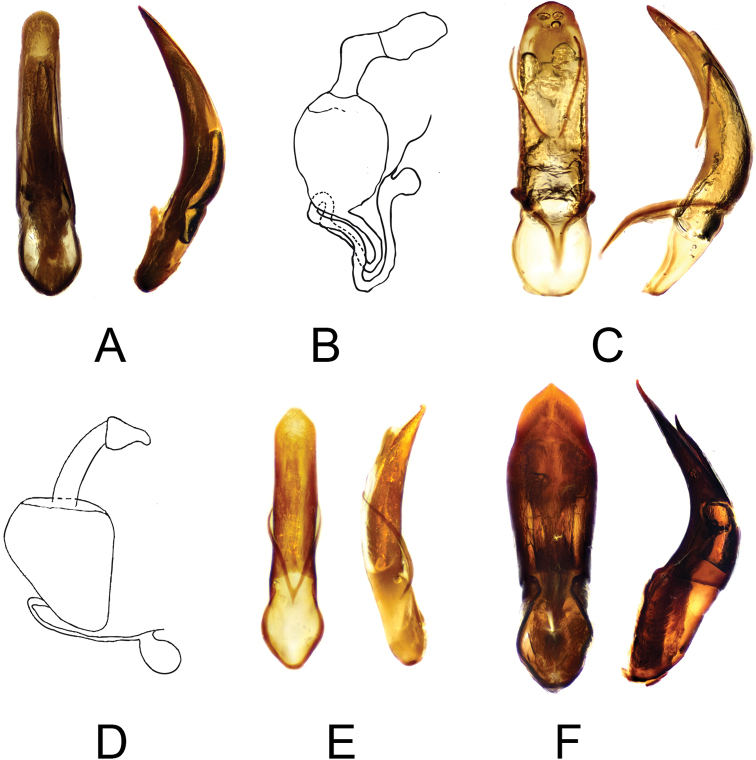
Genitalia *Cangshanaltica luzonica* (**A** aedeagus, **B** spermatheca); *C. mindanaoensis* (**C** aedeagus, **D** spermatheca); *Ivalia antennata* (**E** aedeagus); *I. caligulata* (**F** aedeagus).

#### Description.

***Habitus*.** Body round, convex, 1.9 mm long, 1.4 mm wide, 1 mm high. Colour of ventral and dorsal surfaces chestnut brown, pronotum, head, and antennae somewhat darker than elytra.

***Head*** nearly hypognathous. Frontal calli developed, but indistinctly delimited; surrounded by wide sulcus dorsally. Supraorbital, orbital and suprafrontal sulci developed, indistinct, wide. Frontal ridge wide, feebly elevated; frons short, nearly impunctate. Clypeus straight, developing sharp lateral edges. Antennae short, with 11 antennomeres. Antennomere I bulbous, shorter than antennomeres II and III combined. Antennomere II elliptical, antennomere III feebly elongated. Antennomere IV strongly shortened; antennomeres V–XI pilose, short and widened, forming an elongated antennal club. Antennomeres VI–X darkened.

***Thorax*.** Pronotum strongly convex, twice as broad as long, feebly punctured by small, indistinct, scattered punctures. Anterolateral pronotal setiferous pore placed in the anterior half of the pronotal margin; anterior pronotal margin forming a distinct lobe; posterior pronotal edges widely sharp. Scutellar shield small, triangular. Elytra convex, bearing strong and deep irregularly distributed punctures. Pro- and meso-femora and tibiae feebly pilose, metafemora nearly without setae. Metatibiae strongly curved laterally. Metatarsus attached to metatibia slightly before its end. Metatarsomere I feebly elongated.

***Abdomen*.** Ventrites II–V with a distinct row of setiferous punctures.

***Genitalia*.** Aedeagus moderately curved in lateral view; simple, slender in ventral view, with a feebly distinct step-like narrowing in its apical half. Apex of aedeagus long arrow-like, dull pointed. Female spermatheca unknown.

#### Etymology.

The species name refers to the specific club-like shape of its antennae.

#### Biology.

**Figure 4.**
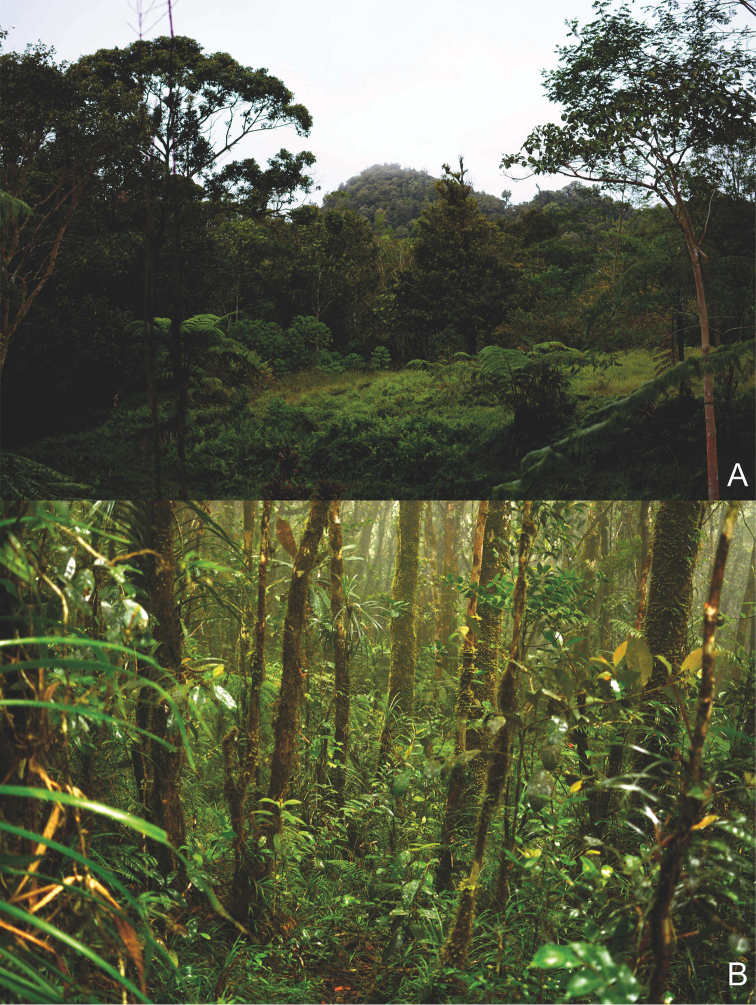
Type localities of *Cangshanaltica mindanaoensis* and *Ivalia antennata***A** montane mossy cloud forest, Mt. Hamiguitan **B** montane forest, Mt. Malambo.

The only known specimen was collected in a montane forest of Mt. Malambo (Fig. 4B) by sifting moss cushions and surrounding leaf litter. The species is likely moss-inhabiting, but we did not perform the gut dissection.

#### DNA barcode sequence.

GenBank accession number: MT654528.

### Ivalia caligulata sp. nov.

Taxon classificationAnimaliaColeopteraChrysomelidae

464DCB38-2B26-5A81-8809-85FDA8782EB9

http://zoobank.org/07FBA761-2A2E-4794-BC28-7469851F82A0

Figures 2B , 3F

#### Type locality.

Philippines: Luzon – Doline NE Sagada.

#### Type material.

***Holotype*** ♂ (MHNG): “Philippines: Luzon. Doline NE Sagada, 21. ii.79 Deharveng-Orousset.” ***Paratypes***: 1 ♂ (NMPC): “Philippines: Luzon. Mount Data, 8.I.80 Deharveng-Orousset.”

#### Generic assignment and differential diagnosis.

The species assigned to *Ivalia* based on the following characters: (1) lack of metathoracic wings and humeral calli; (2) metaventrite bearing a horseshoe-like process reaching mesocoxae; (3) convex body. The species lacks some characters typical for the majority of *Ivalia* species, especially the round body shape and the metatibiae curved laterally. However, there are species assigned to *Ivalia* which are externally similar to this species, e.g., *I. biasa* Takizawa & Konstantinov, 2018, *I. besar* Takizawa & Konstantinov, 2018, *I. fulvomaculata* Takizawa & Konstantinov, 2018 and *I. kinabalensis* Takizawa & Konstantinov, 2018. The new species can be separated from these four species by aedeagus strongly widening towards the apex and having a pointed apex (all mentioned species have aedeagus slender or less widened towards the apex, and the aedeagus apex is dull). The new species also differs from *I. kinabalensis*, *I. biasa*, and *I. besar* in nearly impunctate elytra (moderately to strongly punctate in the latter species). *Ivalia caligulata* also differs from all mentioned species by having a unique shape of pro- and meso-tarsi in males: widened, flat, and elongate, with a strongly pilose ventral side. The form of the metaventral process is somewhat similar to that in *Ivalia korakundah* Duckett et al., 2006; however, the general body shape and coloration is entirely different in both species.

#### Description.

***Habitus*.** Body oblong-ovate, 3.1 mm long, 2.2 mm wide, 1.4 mm high. Head and pronotum pitchy black, elytra black with three wide yellow spots on each elytron. Dorsal surfaces and legs black. Antennae black with antennomeres I–III and XI yellow.

***Head*** nearly hypognathous, triangular. Vertex impunctate, frontal calli feebly delimited, not strongly projecting, triangular. Supraantennal, supraorbital, and orbital sulci deep, suprafrontal sulcus forming a sharp angle delimitating frontal calli. Frontal ridge moderately projecting. Clypeus wide, rounded, feebly and widely incised. Antennae long, with 11 antennomeres. Antennomere I long and bulbous, antennomere II elliptical; antennomeres III–XI generally slender and elongated, feebly widening apically.

***Thorax*.** Pronotum rectangular, convex. Anterolateral pronotal setiferous pore placed apically, anterolateral pronotal angle forming a feeble lobe. Posterior pronotal angles sharp. Elytra convex, nearly impunctate. Legs long; pro- and meso-tarsomere I rectangular, strongly widened, flattened and elongated, densely pilose on ventral side. Mesotibiae slightly curved laterally, flattened. Metatibiae only slightly curved laterally, metatarsomere I elongated; longer than the remaining parts of metatarsus. Metaventrite forming a horseshoe-like anterior process reaching mesocoxae, covering only posterior part of mesoventrite; anterior part of mesoventrite visible.

***Abdomen*.** Ventrite I bearing a long, slender anterior process reaching metacoxae; with a distinct elevated ridge not reaching the rest of the ventrite.

***Genitalia*.** Aedeagus strongly sclerotised, broadly thickened in lateral view, strongly widening towards apex in ventral view. Apex of aedeagus paddle-like, pointed. Female spermatheca unknown.

#### Etymology.

The species name is derived from *caligula* (small shoe in Latin), referring to the widened pro- and mesotarsi of the species.

#### Biology.

Unknown.

### Ivalia postfasciata

Taxon classificationAnimaliaColeopteraChrysomelidae

(Chen, 1934) comb. nov.

73B81B9B-F108-525D-93E7-67FC6B8BEA2C

Figure 2C

Chabria postfasciata Chen, 1934: 399, 416 (type locality: Luzon)

#### Material examined.

***Paratype*** 1 spec. (IZCAS): Luzon.

#### Remarks.

The generic placement of this species is revised based on the following characters: (1) lack of metathoracic wings and humeral calli (*Chabria* species are usually winged and with developed humeral calli); (2) metaventrite forming an anterior horseshoe-like process reaching mesocoxae and covering posterior parts of mesoventrite (the metaventrite of *Chabria* species is simple, without a horseshoe-like process); (3) metatibiae curved laterally (metatibiae not curved in *Chabria*).

#### Redescription.

***Habitus*.** Body oval, convex, 2.4 mm long, 2 mm wide, 1.7 mm high. Head and pronotum black without metallic lustre, elytra black with large orange spots in humeral area and round orange spots in apical area. Legs black with bases of metatibiae and tarsi brown-orange. Antennae black with antennomeres I–II and XI orange. Ventral surfaces dark brown to black.

***Head*** nearly hypognathous, triangular. Supraantennal and orbital sulci deep. Frontal calli feebly developed, elliptical, surrounded by shallow sulci dorsally. Vertex feebly punctate; frontal ridge wide, feebly projecting. Clypeus bearing one row of short setae. Antennae with 11 antennomeres. Antennomere I bulbous, barely shorter than antennomeres II and III combined. Antennomere II elliptical, shortened; antennomeres III–XI long, not strongly widened, antennomeres IV–XI moderately pilose.

***Thorax*.** Pronotum convex, twice as wide as long; impunctate. Anterior pronotal edge feebly forming a lobe, anterolateral pronotal setiferous pore placed in the anterior part of the pronotal margin. Scutellar shield short, wide, triangular. Elytra convex; impunctate. Legs long, 1 and 2 leg pairs moderately pilose. Metatarsomere I strongly elongated, longer than rest of metatarsus; metatibial apical spine longer than metatarsomere II. Metaventral horseshoe-like process reaching mesocoxae; dull, deeply excavated; brown.

***Abdomen*.** Because of the specimen state, we were not able to study the abdomen in detail.

***Genitalia*** were not studied due to the IZCAS rules on handling type specimens.

#### Biology.

Unknown.

##### A checklist of flea beetles from moss-inhabiting genera known from the Philippines with their type localities

***Benedictus* Scherer, 1969**

*B. luzonicus* Sprecher-Uebersax, Konstantinov, Prathapan & Doeberl, 2009 – Luzon (Mt. Data).

***Cangshanaltica* Konstantinov, Chamorro, Prathapan, Ge & Yang, 2013**

*C. luzonica* sp. nov. – Luzon (Sagada env.).

*C. mindanaoensis* sp. nov. – Mindanao (Mt. Hamiguitan, Mt. Malambo).

***Ivalia* Jacoby, 1887**

*I. antennata* sp. nov. – Mindanao (Mt. Malambo).

*I. caligulata* sp. nov. – Luzon (Doline NE Sagada; Mt. Data).

*I. postfasciata* (Chen, 1934) – Luzon.

## Discussion

### Moss-inhabiting flea beetle fauna in the Philippines

Our discovery of three additional *Ivalia* and two additional *Cangshanaltica* species from the Philippines extends the known range of both genera to the Philippines. The *Cangshanaltica* species described here represent the first known *Cangshanaltica* from humid equatorial tropics. Both species are very similar and may be closely related; they may be part of a possibly existing radiation in the Philippine archipelago and its mountain ranges. We expect that more species of *Cangshanaltica* do occur in different islands or mountain ranges. In *Ivalia*, the Philippine species differ greatly from each other, and we hence do not expect them to be closely related. *Ivalia caligulata* strongly resembles several species described from Mt. Kinabalu, Borneo, possibly indicating that *I. caligulata* is a Sundean faunal element. Relationships of the other two *Ivalia* species described here cannot be assumed based on the morphology. Molecular grade material is needed to test the above hypotheses and understand the origin and biogeography of both genera in the Philippines. Additional material is also needed from islands other than Luzon and Mindanao, especially from the Visayas, and from additional mountain ranges. The list of species presented here is very preliminary and many more moss-inhabiting species may be expected, which is also clearly visible on the distributional map of known moss-inhabiting flea beetles in the Philippines (Fig. 6).

### Club-like antennae in moss-inhabiting flea beetles

**Figure 5.**
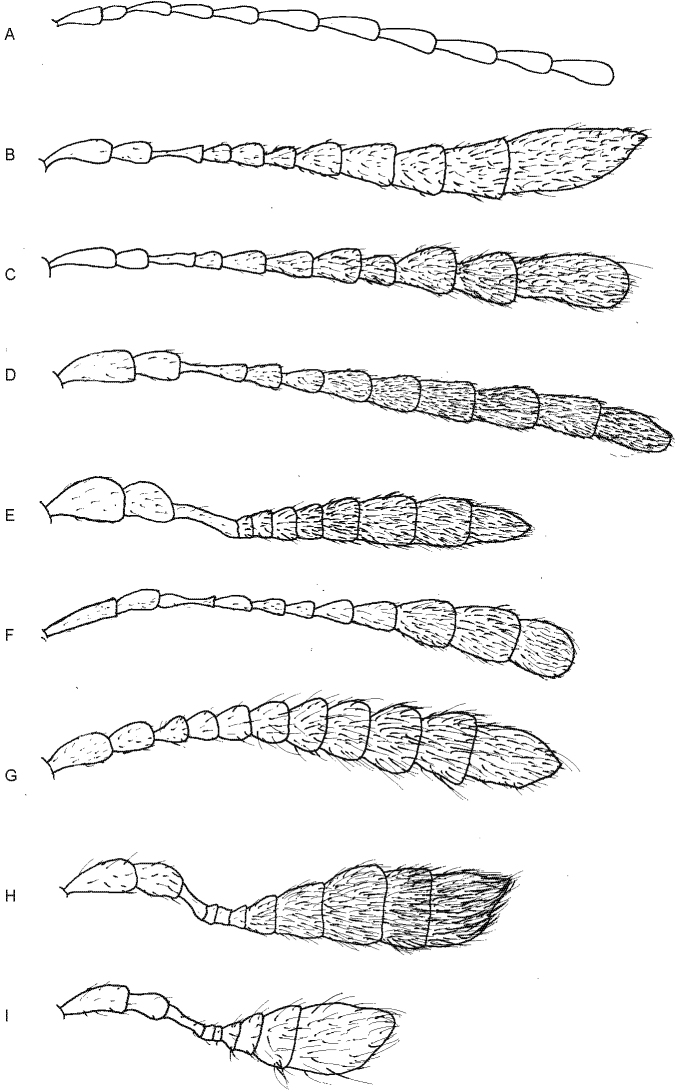
Antennae of various genera and species of moss-inhabiting flea beetles showing various level of antennal club formation **A***Ivalia besar***B***Ivalia lescheni***C***Ivalia iridescens***D***Ivalia uenoi***E***Ivalia antennata***F***Mniophilosoma laeve***G***Borinken elyunque***H***Clavicornaltica doeberli***I***Kiskeya baorucae*.

Some of the newly described species of *Ivalia* show unique morphological characters and suggest morphological trends, which were never discussed before. *Ivalia antennata* has strongly thickened antennae, forming a long, but distinct antennal club. Among known leaf litter and moss-inhabiting flea beetles, fully formed club-like antennae are known only in genera with the strongest morphological specialisation, including also extremely compact body: *Kiskeya* Konstantinov et al., 2009 found in the Neotropics, and *Clavicornaltica* Scherer, 1974, a highly diverse, but enigmatic Oriental genus (Scherer 1974, Konstantinov and Duckett 2005, Konstantinov et al. 2009). Somewhat club-like antennae are also present in various moss-inhabiting flea beetles from the mainly Neotropical *Monoplatus* group, e.g., in *Distigmoptera* Blake, 1943 (Konstantinov and Konstantinova 2011). We do not find a well-formed antennal club in other moss-inhabiting genera; however, we can usually observe at least thickened apical antennal segments, suggesting a trend for club-like antennae (Fig. 5). This is e.g., the case of *Cangshanaltica* Konstantinov et al., 2013, *Mniophila* Stephens, 1831, *Borinken* Konstantinov et al., 2011, *Mniophilosoma* Wollaston, 1854. In *Ivalia*, antennae can be long and filiform (e.g., in *I. caligulata*, *I. besar*, *I. biasa* or *I. kinabalensis*) or more less thickened apically (e.g., *I. uenoi*, *I. korakundah*, *I. lescheni* and *I. iridescens*) (Duckett et al. 2006, Nadein 2013, Takizawa and Konstantinov 2018). *Ivalia antennata* described above has antennal club even more developed, with shortened antennomeres on its basal part, unlike any other *Ivalia* known at the moment. It seems that evolution of the antennal club is a more complex process than the evolution of the compact body, flightlessness, or convex body shape typical for the majority of moss-inhabiting flea beetles.

**Figure 6:**
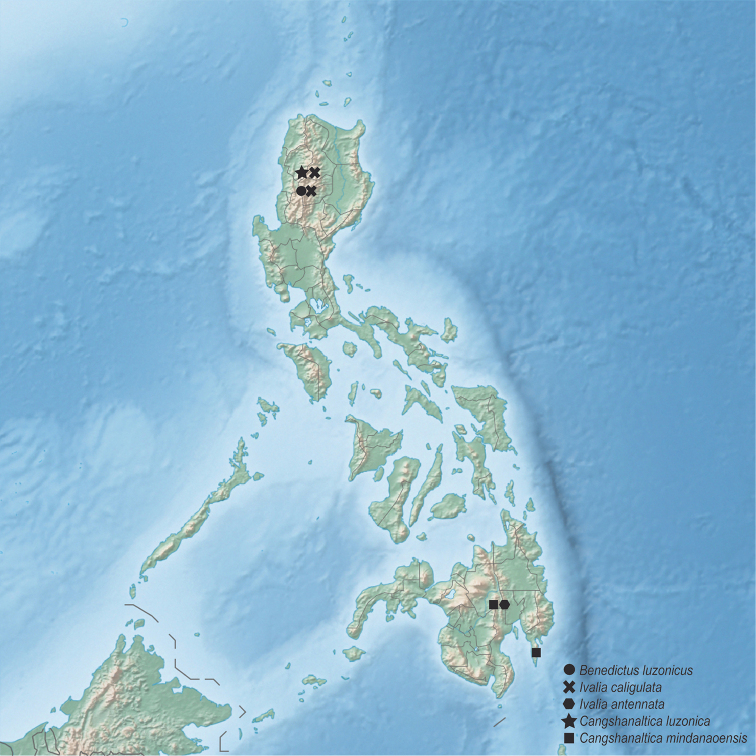
Current knowledge about distribution and diversity of moss-inhabiting flea beetles in the Philippines.

### Comments to future taxonomic work on moss-inhabiting flea beetles

The finding that *Chabria postfasciata* belongs to the genus *Ivalia* indicates that some moss-inhabiting flea beetle species may have been described for a long time but misplaced in other genera, with types hidden in museum collections and never re-examined. Old museum collections also hide vast numbers of undescribed moss-inhabiting flea beetle species. For example, the recently described *Adamastoraltica*, a flightless flea beetle from Africa, was also found in an older collection (Biondi et al. 2020). This study is based on older museum material as well as the newly collected material, which proved to be the ideal approach. Systematic revisional work of ecologically specialised and largely unknown groups should focus not only on field work but on examining forgotten material in museum collections.

## Supplementary Material

XML Treatment for Cangshanaltica

XML Treatment for Cangshanaltica luzonica

XML Treatment for Cangshanaltica mindanaoensis

XML Treatment for Ivalia

XML Treatment for Ivalia antennata

XML Treatment for Ivalia caligulata

XML Treatment for Ivalia postfasciata
